# Treatment outcomes of symptomatic internal iliac artery aneurysms

**DOI:** 10.4314/gmj.v59i1.7

**Published:** 2025-03

**Authors:** Lily P Wu, Jessica Dei-Asamoa, Olutobi A Sanuade

**Affiliations:** 1 School of Medicine and Dentistry, University of Ghana Medical School; 2 Korle Bu Teaching Hospital, Accra, Ghana; 3 Department of Population Health Sciences, Division of Health System Innovation and Research, Spencer Fox Eccles School of Medicine at the University of Utah, Salt Lake City, Utah, USA

**Keywords:** Iliac aneurysm, surgical treatment outcomes, pelvic pain, open surgical repair, Ghana

## Abstract

Isolated internal iliac artery aneurysm (IIIAA) is a rare condition, with limited information available about its symptoms and surgical treatment outcomes, particularly in Ghana. This case report presents the 4-year surgical treatment outcomes of three patients (aged 42, 58, and 22 years) with IIIAA, each exhibiting different clinical presentations. All patients experienced pelvic pain and difficulty walking but had uneventful post-operative recoveries. They made significant progress with physiotherapy and were able to mobilise with a Zimmer frame before discharge. Currently, all three patients are mobilising unaided, and post-operative Computed Tomography (CT) scans show no complications. This report highlights the importance of early diagnosis to minimise complications and improve the prognosis for IIIAA, which often presents asymptomatically and can manifest with a wide range of clinical findings. The study underscores the effectiveness of open surgical repair in achieving positive outcomes for IIIAA patients in Ghana.

**Funding:**

## Introduction

Most iliac artery aneurysms are associated with abdominal aortic aneurysms (AAA). Up to 10-15% of AAA co-exist with iliac artery aneurysm.^1^ Iliac artery aneurysm (IAA) is a dilatation of the common, internal, or external iliac arteries or various combinations of them, without the involvement of the infra-renal abdominal aorta. Isolated internal iliac artery aneurysms (IIIAA) are uncommon, making up 0.3-0.5% of all intra-abdominal aneurysms. IIIAA is defined as focal dilatation of the internal artery alone with a threshold for surgical treatment set at 3.5 cm.^1^,^2^

IIIAA is a disease of the elderly (aged 65-75 years) with a male-to-female ratio of between 5-16:1.^2^,^3^ Risk factors and aetiologies associated with Isolated Internal Iliac artery aneurysms include infection (viral and bacterial), trauma and arterial wall disorders due to connective tissue diseases like Marfan and Ehlers-Danlos syndrome etc. However, the predominant aetiological factor is degeneration, hence its predilection for the aged. Some organisms responsible for the infective causes of IIIAA are Salmonella spp., Staphylococcus aureus, Escherichia coli and Tuberculosis.^4^ Infective causes of IIIAA is most often found in relatively young people below the age of 50 years.

Symptom manifestation of IIIAA is usually late and include pelvic pain from nerve root compression or pressure on surrounding structures. Other symptoms include pyelonephritis from urosepsis secondary to ureteric obstruction which can also manifest at flank pain. IIIAA is an uncommon cause of deep vein thrombosis (DVT).^5^,^6^ We report the surgical treatment outcomes of three cases of IIIAA, all male patients presenting with different complications of IIIAA. Written informed consent was obtained from the patients for publication of this case report and accompanying images.

## Case Presentation

### Case 1

A 42-year-old tailor presented with severe left hydronephrosis, hydroureter, and a non-functional left kidney. A CT angiogram revealed a large fusiform left internal iliac aneurysm (8.9 cm). The patient, a nonsmoker with no history of hypertension, hypercholesterolemia, or connective tissue disease, experienced severe pelvic pain and was unable to walk for two months prior to his clinic visit. The laboratory and radiological examinations performed are shown in Table 1.

**Table 1:** Laboratory and radiological examinations performed and their corresponding findings for the patients

Tests	Case 1 Findings	Case 2 Findings	Case 3 Findings
**FBC**	HB 11.4 g/dL (10-14), WBC 10.46 x 10^9/L (2.50-8.50), PLT 338 x 10^9/L (150-450)	HB 10.8 g/dL (10-14), WBC 12.07 x 10^9/L (2.50-8.50), PLT 286 x 10^9/L (150-450)	HB 7.9 g/dL (10-14), WBC 13.86 x 10^9/L (2.50-8.50), PLT 438 x 10^9/L (150-450)
**BUE, Cr**	Cr 155.0 µmol/L (71-133), Urea 8.2 mmol/L (2-7), Na 138 mmol/L (135-150), K 5.5 mmol/L (3.5-5.5)	Cr 105.0 µmol/L (71-133), Urea 6.2 mmol/L (2-7), Na 138 mmol/L (135-150), K 5.0 mmol/L (3.5-5.5)	Cr 115.0 µmol/L (71-133), Urea 11.2 mmol/L (2-7), Na 122 mmol/L (135-150), K 5.5 mmol/L (3.5-5.5)
**Hepatitis B and C**	Negative	Negative	Negative
**HIV screen**	Negative	Negative	Negative
**Syphilis screen**	Negative	Negative	Negative
**Blood culture**	No bacteria growth	No bacteria growth	No bacteria growth
**Chest X-ray**	Normal	Normal	Normal
**ECG**	Normal	Normal	Normal
**Echocardiogram**	Normal	Normal	Normal
**Computerized tomography venogram (CTV)**	8.9 cm left IIAA, severe hydronephrosis, hydroureter, non-functional left kidney	7.8 cm left IIAA, left hydronephrosis, hydroureter, functional left kidney	8 cm left IIAA, ilio-femoral DVT

On examination, he appeared acutely ill with a Body Mass Index (BMI) of 24.1 kg/m^2^. His vital signs included a blood pressure of 128/78 mmHg, a pulse rate of 98 bpm, and a temperature of 36.8°C. His left lower limb was held in flexion and external rotation at the hip, appearing larger than the right limb. Measurements showed a mid-thigh circumference of 45 cm, calf circumference of 32 cm, and ankle circumference of 22 cm on the left, compared to 40 cm, 30 cm, and 20.5 cm on the right. Peripheral pulses were present in both lower limbs but were weaker on the left. The left lower limb was tender with intact sensation but reduced motor function (3/5).

The patient was admitted and treated with intravenous (IV) antibiotics (amoxicillin clavulanic acid and ciprofloxacin) for four days pre-operatively and continued post-operatively for two weeks. Pain management included IV paracetamol and intramuscular pethidine. Pre-operative work-up and imaging were conducted (Figure 1a). Definitive surgery involved an open primary repair with a total nephrectomy due to the non-functional kidney (Figure 2).

**Figure 1a:**
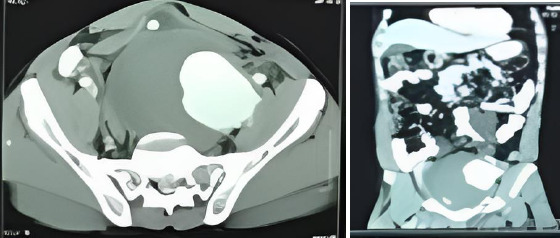
Pre-operative CT angiogram of Case 1

**Figure 1b:**
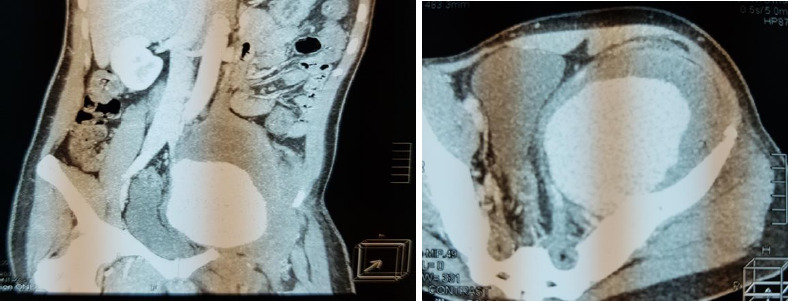
Pre-operative CT angiogram of patient 2

**Figure 1c:**
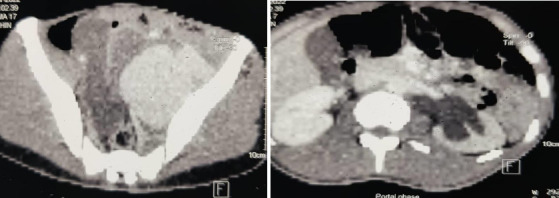
Pre-operative CT angiogram of patient 3

**Figure 2:**
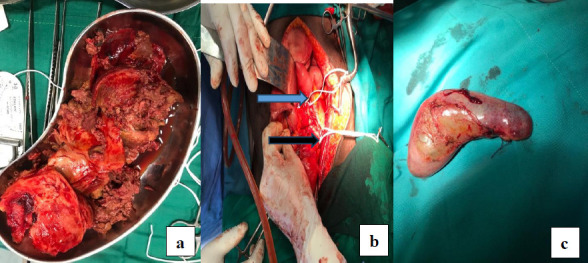
intra-operative pictures of patient 1 showing laminar thrombus in a kidney dish (a), aneurysmal sac (b) with a proximal sling on CIA (blue arrow) and distal sling on EIA (black arrow) and a non-functional left kidney post nephrectomy(c) sample

The peritoneal cavity was explored through a midline incision, and the aneurysm was identified. The left common iliac (CIA) and external iliac arteries (EIA) were mobilised and clamped after administering IV heparin. The aneurysm was opened, the thrombus was evacuated, and the aneurysmal sac was excised. The left internal iliac artery (IIA) stump was ligated, and the distal branches were sutured. The total operating time was three hours, with an estimated blood loss of one litre. The patient received two units of whole blood and two units of fresh frozen plasma intra-operatively.

Postoperatively, the patient had an uneventful recovery, with an 18-day hospital stay. On postoperative day eight, antibiotics were switched to oral, and the Pneumovac was removed on day ten. He made good progress with physiotherapy, mobilising with a Zimmer frame before discharge. His haemoglobin was 10.1 g/dL at discharge, with normal full blood count and renal function.

Follow-up at two weeks and three months post-discharge showed he was doing well. Now, three years post-operative, he mobilises unaided, and post-operative CT scans show no complications.

### Case 2

A 58-year-old male from Liberia was referred to Ghana to see a Vascular Surgeon due to difficulty walking, pelvic pain, and a CT angiogram finding of a 7.8 cm fusiform left internal iliac aneurysm with left hydronephrosis and hydroureter. He was a known hypertensive but well-controlled on anti-hypertensive medication.

On examination, he appeared acutely ill with a BMI of 23.8 kg/m^2^. His blood pressure was 145/84 mmHg, pulse rate 86 bpm, and temperature 37.2°C. His left lower limb was held in flexion and external rotation at the hip, appearing larger than the right limb. Measurements showed a mid-thigh circumference of 47.5 cm, calf circumference of 34 cm, and ankle circumference of 22.5 cm on the left, compared to 41.5 cm, 31 cm, and 21.5 cm on the right. Peripheral pulses were present in both lower limbs but were weaker on the left. The left lower limb was tender with intact sensation but reduced motor function (2/5).

**Figure 3:**
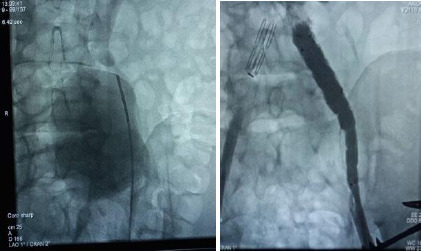
intra-operative on table angiogram of patient 2 showing a large left IIA, aorto-uni-iliac stent, right CIA plug and a fem-fem crossover

The patient was admitted and treated with IV antibiotics (amoxicillin clavulanic acid and ciprofloxacin) for four days pre-operatively and continued post-operatively for one week. Pain management included IV paracetamol and intramuscular pethidine. Pre-operative work-up and imaging were conducted (Figure 1b). He underwent an endovascular repair with a hybrid operation involving plugging of the right common iliac artery, insertion of an aorto-uni-iliac stent graft on the left, and a left-to-right fem-fem crossover with a reinforced 8mm PTFE graft (Figure 3).

Post-operatively, the patient had an uneventful recovery, with a hospital stay of 15 days. Antibiotics were switched to oral on the first postoperative day. He made good progress with physiotherapy, mobilising with a Zimmer frame before discharge. His haemoglobin was 10.7 g/dL at discharge, with normal full blood count and renal function. Follow-up at two weeks and three months post-discharge showed he was doing well. Now, two years postoperative, he mobilizes unaided, and post-operative CT scans show no complications. He is currently on surveillance with serial CTA every six months and reviewed electronically via Zoom or WhatsApp.

### Case 3

A 22-year-old male was referred to the Vascular Unit with a diagnosis of left deep vein thrombosis (DVT), confirmed by a venous duplex scan. Despite being treated with anticoagulation (IV unfractionated heparin) for two weeks at another hospital, his condition worsened, leading to increased pain, swelling, and immobility in his left leg. He had no known risk factors for DVT, no family history, and was a non-smoker. A CT angiogram revealed an 8cm fusiform aneurysm in the left internal iliac artery, compressing the left external iliac vein and causing ilio-femoral DVT (Figure 1c).

**Figure 4:**
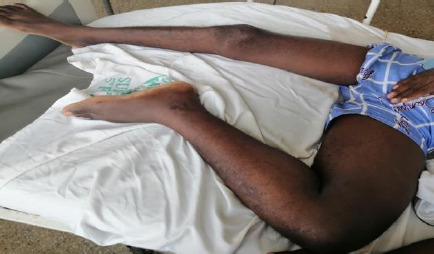
A picture of patient 3 pre-operative showing limb posture and circumference discrepancy

On presentation, he was acutely ill, with a body mass index (BMI) of 23.2kg/m^2^, a blood pressure of 134/92mmHg, and a pulse rate of 115 beats per minute. His left lower limb was significantly larger than the right. On the left lower limb, his mid-thigh circumference was 48cm, his calf circumference was 35cm and his ankle circumference was 23.5cm compared to 39cm, 30cm and 20cm respectively on the right. The left lower limb was tender; sensation was intact but motor function was reduced (2/5) (Figure 4).

He was admitted and treated with antibiotics (IV amoxicillin clavulanic acid and IV ciprofloxacin) for four days before surgery and one week post-operatively. The pain was managed with IV paracetamol and intramuscular pethidine. Pre-operative investigations included a transfemoral insertion of an IVC filter to prevent embolisation.

**Figure 5:**
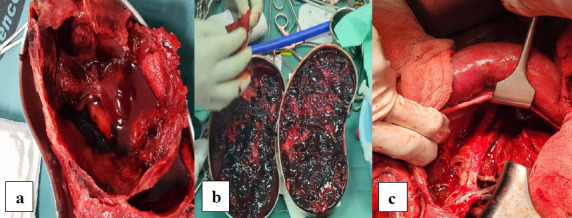
Intra-operative pictures of Case 3 showing laminar thrombus (a), aneurysmal sac in a kidney dish (b) and the remaining cavity in the left iliac fossa (c)

The surgical procedure involved a midline laparotomy to access the aneurysm, which was excised after clamping the common iliac and external iliac arteries. The defect in the common iliac artery was repaired with 5.0 Prolene, and an iatrogenic injury to the external iliac artery was also repaired with 6.0 Prolene (Figure 5).

The peritoneal cavity was lavaged with warm saline, and a Pneumovac was inserted for drainage. The surgery lasted six hours, with an estimated blood loss of 3 litres. The patient received blood and plasma transfusions intra-operatively.

**Figure 6:**
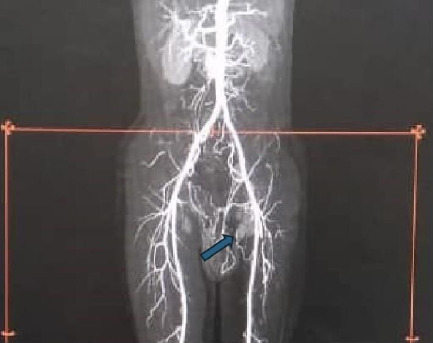
Postoperative computerized tomography of patient 3 showing successful aneurysmal excision and patent left iliac arteries. (NB: Arrow points to left internal iliac artery stump)

Post-operatively, the patient recovered well. He was switched from IV to oral antibiotics and made significant progress with physiotherapy, mobisliing with a Zimmer frame before discharge. His hospital stay lasted 20 days, and by discharge, his haemoglobin level was 10.4g/dl, with normal blood counts and renal function. At follow-up visits two weeks and three months later, he was doing well. Three years post-operation, he is fully mobile without assistance, and post-operative CT scans show no complications or adverse events (Figure 6).

## Discussion

This study is the first in Ghana to report symptoms and surgical outcomes of isolated iliac artery aneurysms (IIIAAs). We examined three male patients, aged 22–58, who presented with complications of IIIAA. Our findings emphasize the importance of timely diagnosis and prompt surgical intervention for IIAA complicated by progressive deep vein thrombosis (DVT). This study also aims to raise awareness among health professionals about the potential for positive outcomes in IIAA cases in Ghana if proper guidelines are followed.

Even though evidence shows that IIAA is a relatively uncommon vascular disease, especially among young people,^7^ all the patients in this study were below 65 years old. This suggests a possibility of an earlier onset of IIIAA in Ghana. Previous studies have noted that IIIAA is more prevalent in males,^7^ which aligns with our study, but further research is needed to explore why this condition disproportionately affects men.

Clinically, all three patients presented with pelvic pain and difficulty walking, symptoms commonly associated with IIAA from nerve compression and pressure on surrounding structures.^8^ Other clinical presentations included left internal iliac aneurysm, left leg DVT, and left hydronephrosis and hydroureter. While urinary tract infections (UTIs) are a known complication of IIIAA, none of the patients in this study had UTIs. However, DVT, as seen in patient 3, has been previously documented as a complication.^9^

All patients had larger left lower limbs with reduced motor function, consistent with research showing that over 61% of IIIAA cases affect the left side.^7^,^10^ None had significant atherosclerosis risk factors, highlighting the atypical nature of these cases. As the internal iliac artery is deep in the pelvis, previous research showed that most IIIAAs show no symptoms until they become large or rupture.^2^ This shows the need for early diagnosis and effective management strategies to prevent severe outcomes.

All three patients underwent open surgical repair, which is one of the standard treatment options for IIIAA alongside endovascular repair and hybrid techniques.^7^ Despite the high mortality risk associated with untreated IIIAA, 8,9 all patients in this study had favourable outcomes. They were discharged after an average 14-day hospital stay, fully mobilised, and had no post-operative complications, as confirmed by CT scans. These results underscore the effectiveness of open surgical repair for managing IIIAA in Ghana.

## Conclusion

IIIAA is a low-incidence disease that is often asymptomatic and can present with a wide range of clinical findings. Treatment with open surgical repair is effective in getting positive outcomes in asymptomatic patients. Since patients usually present with no symptoms, the need for early diagnosis to minimize complications and worse prognosis is important. Also, further research is needed to explore the reasons for the higher prevalence of IIAAs among men as well as relatively younger people in Ghana.
